# Analysis of Large Scale Spatial Variability of Soil Moisture Using a Geostatistical Method

**DOI:** 10.3390/s100100913

**Published:** 2010-01-25

**Authors:** Tarendra Lakhankar, Andrew S. Jones, Cynthia L. Combs, Manajit Sengupta, Thomas H. Vonder Haar, Reza Khanbilvardi

**Affiliations:** 1 NOAA–Cooperative Remote Sensing Science & Technology Center, (NOAA-CREST), City University of New York, NY 10031, USA; E-Mail: khanbilvardi@ccny.cuny.edu; 2 Cooperative Institute for Research in the Atmosphere (CIRA), Colorado State University, Fort Collins, CO 80523, USA; E-Mails: jones@cira.colostate.edu (A.S.J.); combs@cira.colostate.edu (C.L.C.); vonderhaar@cira.colostate.edu (T.H.V.H.); 3 National Renewable Energy Laboratory, Golden, CO 80401, USA; E-Mail: Manajit.Sengupta@nrel.gov

**Keywords:** soil moisture, kriging, variogram, AGRMET, Oklahoma Mesonet

## Abstract

Spatial and temporal soil moisture dynamics are critically needed to improve the parameterization for hydrological and meteorological modeling processes. This study evaluates the statistical spatial structure of large-scale observed and simulated estimates of soil moisture under pre- and post-precipitation event conditions. This large scale variability is a crucial in calibration and validation of large-scale satellite based data assimilation systems. Spatial analysis using geostatistical approaches was used to validate modeled soil moisture by the Agriculture Meteorological (AGRMET) model using *in situ* measurements of soil moisture from a state-wide environmental monitoring network (Oklahoma Mesonet). The results show that AGRMET data produces larger spatial decorrelation compared to *in situ* based soil moisture data. The precipitation storms drive the soil moisture spatial structures at large scale, found smaller decorrelation length after precipitation. This study also evaluates the geostatistical approach for mitigation for quality control issues within *in situ* soil moisture network to estimates at soil moisture at unsampled stations.

## 1. Introduction

Spatial characteristics of soil moisture dynamics is essential in the hydrological and meteorological modeling, improves our understanding of land surface–atmosphere interactions. Spatial and temporal soil moisture dynamics are essential to improve the parameterization for agricultural, hydrological and meteorological modeling processes. Knowledge of spatial and temporal soil moisture variability is required for calibration and validation of satellite based soil moisture products [1,2]. Spatial distribution of soil moisture directly affects the incident precipitation into runoff over complex terrain. The hydrological modeling processes are based on spatially lumped conceptual models needs realistic representation of lateral distribution of soil moisture [1]. Therefore, current research needs to be focused on assimilation of spatial and temporal dynamic of soil moisture including remote sensing data in to the forecast models to improve accuracy [3–6].

Soil moisture spatial distribution varies both vertically and laterally due to evapotranspiration and precipitation, influenced by topography, soil texture, and vegetation. While small scale spatial variations are influenced by soil texture, larger scales are influenced by precipitation and evaporation [7]. The characteristics of soil moisture variability is essential for understanding and predicting land surface processes, that varies based on topography, soil texture, and vegetation at different spatial and temporal scales [8]. Thus, the spatial characteristics is a key parameter used in the background statistical error models as well dynamic propagation of the modeled state uncertainty in data assimilation modeling systems [5,9–12].

Spatial characteristics in terms of horizontal decorrelation lengths determine the sharing of information within cost function of data assimilation system, directly impacts the system performance. Without this information, educated guesses are typically employed for the horizontal scale lengths [9]. This can be result in two possible data assimilation behavior errors: (1) an under-estimate of the horizontal decorrelation length scale resulting in unrealistic decoupling of the data assimilation spatial effects, thus needlessly increasing the data assimilation system errors thereby requiring a stronger remote sensing signal strength to compensate for the errors, and (2) an over-estimate of the horizontal decorrelation length scale resulting in overly smooth data assimilation output results resulting in loss of high resolution soil moisture information available from the data. Thus, accurate spatial correlation scale information minimizes the loss of data assimilation accuracy and data signal strength.

**Figure 1.**
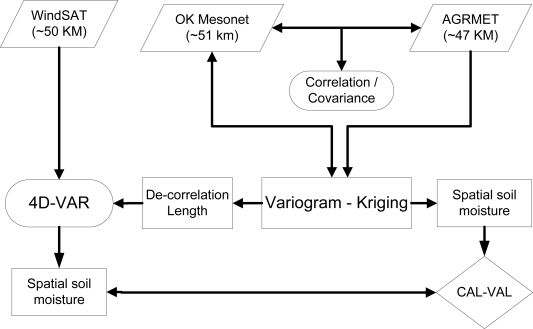
Application of variogram and kriging analysis in calibration and validation of soil moisture information for data assimilation process.

The characteristic of spatial variability of soil moisture depends on the scale of observation. Previous geostatistical studies were carried out to understand spatial and temporal soil moisture dynamics at the scales of small (1–5 km^2^) catchment areas [13–18]. However, areal extent of these studies is too small for robust soil moisture analysis at precipitation scales as well as spatial scales (20–50 Km) of soil moisture retrieval from passive microwave satellite data. The spatial extent (or footprint) of AGRMET and satellite based passive microwave radiometers is comparable to average distance between two Oklahoma Mesonet soil moisture sensors. This distance is approximately equal to precipitation storm-scales, which drive the soil moisture spatial structures [19], therefore the knowledge of the spatial structure at these relatively crude resolutions is needed with the increase in availability large scale remote sensing satellite radiometer (WindSat, AMSR-E, SMOS, NPOESS) data for soil moisture retrieval. Figure 1 illustrates typical application variogram and kriging analysis to bring new spatial dynamic of soil moisture characteristics into the forecast models to improve hydrological modeling and assimilation results.

This study evaluates *in situ* Oklahoma Mesonet network and Agriculture Meteorology model (AGRMET) soil moisture using spatial analysis. The AGRMET generates satellite-based radiation and precipitation products, which is used as forcing in the Global Land Data Assimilation System [20]. Similarly, Oklahoma Mesonet data are widely used by the research community to validate and improve land surface models used in numerical weather prediction modeling [21].

There are two primary components of this study: (1) use of the variogram technique to provide a large-scale estimate of soil moisture characteristics for the entire state of Oklahoma, and (2) evaluation of the kriging technique to validate modeled (AGRMET) soil moisture data with *in situ* Oklahoma Mesonet soil moisture data.

## 2. Study Area and Data Sets

**Figure 2.**
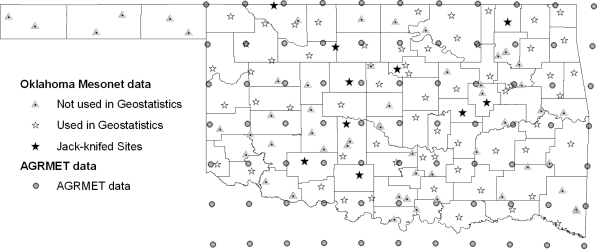
The distribution of AGRMET grid points and Oklahoma Mesonet sites used in the geostatistical analysis.

For this study, soil moisture and precipitation information collected from Oklahoma Mesonet and the AFWA’s AGRMET model during September 2003 were used. The study area covered the entire State of Oklahoma, which has a sub-humid climate with moderately rolling topography. A strong cold-front with associated precipitation crossed the Midwest during September 2003, allowing observation of soil moisture both before and after a heavy rain event, including observations during the drying period. Many Oklahoma Mesonet locations experienced at least two significant rain events. Figure 2 shows the distribution of AGRMET grid points and Oklahoma Mesonet sites used in the study. More details about Oklahoma Mesonet site selection are discussed in Section 3.3.

### 2.1. In Situ Oklahoma Mesonet

The Oklahoma Mesonet is a statewide mesoscale environmental monitoring network consisting of 110 automated stations measuring more than 20 environmental variables with at least one station in each of Oklahoma’s 77 counties [21,22]. This statewide monitoring network was originally set up by Oklahoma State University agricultural scientists to expand the use of weather data in agricultural applications and the needs of University of Oklahoma scientists to plan and implement a flood warning system in Tulsa [21,23]. Since these datasets were not collected with satellite or model calibration in mind, we must understand the strengths and limitations of each dataset in order to properly compare *in situ* measurements with satellite-based or model-based results. Sensor-specific calibration coefficients generated via linear regression were applied to the soil moisture data. Soil moisture sensors were calibrated using driest and wettest observations from laboratory and field sites [21].

Soil moisture is measured at each site at depths of 5 cm, 25 cm, 60 cm and 70 cm at 30 minute time intervals. Since 1994, the Oklahoma Mesonet has collected almost 3.5 billion (10^9^) weather and soil observations [21]. The instruments used in Mesonet Network for soil moisture measurement, are a Campbell Scientific 229-L heat dissipation sensor [23]. This sensor consists of a heating element and thermocouple emplaced in epoxy in a hypodermic needle, which is encased in a porous ceramic matrix. This sensor is typically used to measure soil metric potential by determining the temperature difference of the sensor before and after a heat pulse is introduced. The temperature difference is then converted into Fractional Water Index (FWI) and volumetric soil moisture. For physically-based land surface models, the more quantitative volumetric soil moisture measure is preferable due to mass transport of water within the soil column.

### 2.2. AGRMET Model

The Air Force Weather Agency (AFWA) has been running its AGRMET model operationally in near-real time for use by the Air Force and the U.S. Department of Agriculture to predict global grain production [24]. AGRMET is a near real-time global land surface analysis model that uses the NOAH community land-surface soil hydrology module as its core module. The AGRMET model basically uses satellite remote sensing data to generate spatial hydro-meteorological data and also utilizes WMO ground station data in the algorithms. AGRMET specifies nine soil types and thirteen vegetation types. AGRMET model produces three hourly hydro-meteorological parameters such as soil temperature, soil moisture, snow water equivalent, Canopy and moisture content data at 47 km resolution [24,25]. These datasets used by Global Land Data Assimilation System (GLDAS) to generate global, downward shortwave and longwave radiation fluxes at the land surfaces [20].

AGRMET model uses two polar stereographic grids which covers all major land areas of the northern and southern hemispheres. One of its unique features is that it produces a three hourly Special Sensor Microwave Imager (SSM/I) based rain estimate as one of several sources of estimated precipitation. The AGRMET model uses Richard’s equation to predict soil moisture at four soil layer depths: 0–10 cm, 10–40 cm, 40–100 cm and 100–200 cm. In order to compare model output with *in situ* Oklahoma Mesonet data, a 110 km by 63 km grid box was centered over each site and averaged.

## 3. Methodology

### 3.1. Variogram and Kriging

A variogram, a central concept in geostatistics, is used to analyze the structure of spatial variation of soil moisture. The variogram structure consists of the nugget (the variance at zero lag distance), sill (the variance to which the variogram asymptotically rises), and decorrelation length (range of spatial dependence). The decorrelation length relates to spatial variability of variables estimated based on an experimental variogram. The decorrelation length varies based on minimum distance between sampling locations and size of sampled area [15].

**Figure 3.**
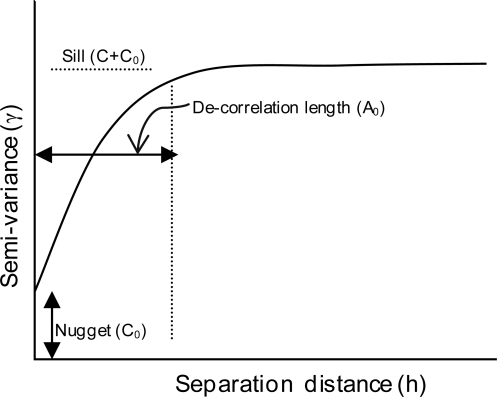
A generalized variogram model shows the essential components: nugget is the y-intercept that represents the semi-variance between two closest points; Sill signifies maximum semi-variance; and decorrelation length measures spatial continuity.

Understanding the variogram helps to relate some of the descriptors of the variogram to the spatial characteristics of the data. The variogram as shown in Figure 3 represents half of the variance between two points in a spatial field as a function of their separation or lag distance [26]. Mathematical models, such as spherical, gaussian, or exponential can be fitted to the experimental variogram for visualization of the variable’s spatial variation. Semi-variance or half of the variance is an autocorrelation statistic defined as [26]:

(1) $$\gamma (h)=\frac{1}{2N(h)}\sum_{i=1}^{N(h)}{\left(z_{i}-z_{i+h}\right)}^{2}$$

where, γ(h) = Semi-variance for interval distance class (h), Z_i_ = measured sample value at point *i*, *Z_i+h_* = measured sample value at point *i + h*, and N (h) = total number of sample couples for the separation interval *h*.

The least squares best-fit criteria is used to fit a model to the experimental semi-variance data through which the nugget (C_0_), sill (C_0_ + C), and decorrelation length or range of spatial dependence (A_0_) (Figure 3) are determined. These parameters of the variogram model describe the characteristics of spatial variation. The nugget is the y-intercept of the variogram indicating the semivariance between the two closest points separated in the spatial field. The sill of the variogram model represents the spatially dependent variance. Theoretically, the sill is equivalent to the maximum semivariance when the variogram model is bounded. The decorrelation length or range measures the limit of dependence of a given variable and is the distance at which the variogram reaches its sill. This is the limit of spatial dependence. If the decorrelation length is large then long-range variations dominate; if it is small, then the major variation occurs over short distances [26].

The most commonly used variogram models namely spherical, exponential, gaussian and linear are defined as follows:

(2) $$\text{Spherical model}:\;\;\;\gamma (h)=C_{0}+C\left[1.5\left(\frac{h}{A_{0}}\right)-0.5{\left(\frac{h}{A_{0}}\right)}^{3}\right]\;\;\;\text{for}\;h\leq A_{0}$$

(3) $$\text{Gaussian model}:\;\;\;\gamma (h)=C_{0}+C\left[1-\text{exp}\left(-\frac{h^{2}}{A_{0}^{2}}\right)\right]$$

(4) $$\text{Exponential model}:\;\;\gamma (h)=C_{0}+C\left[1-\text{exp}\left(-\frac{h}{A_{0}}\right)\right]$$

(5) $$\text{Linear model}:\;\;\;\gamma (h)=C_{0}+\left[h\left(\frac{C}{A_{0}}\right)\right]$$

where:

γ(h) = semivariance for interval distance class *h*;
h = the separation distance interval
C_0_ = nugget variance ≥ 0;
C = structural variance ≥ C_0_; and
A_0_ = decorrelation length or range parameter.

The spherical model is a modified quadratic function for which at some distance A_0_, pairs of points will no longer be autocorrelated and the variogram reaches an asymptote (spherical model effective range A = A_0_). However, in the case of Gaussian or hyperbolic models the sill never meets the asymptote. In such a condition, the effective range (A = √3A_0_) is the distance at which the sill (C_0_ + C) is within 5% of the asymptote. The utility of correlated variables become less useful at lengths beyond their horizontal decorrelation length scales. These measures are typically represented by an exponential length scale decay in their correlations such that the covariance is proportional to exp (–1/A), where A is the decorrelation length.

Kriging is an interpolation technique based on the theory of regionalized variables developed by Matheron [27]. Kriging is an interpolation technique that generates the best linear unbiased estimate at each location using the spatial variability obtained from the variogram model. Kriging offers a wide and flexible variety of tools that provide estimates for unsampled locations using weighted average of neighboring field values falling within a certain distance called the range of influence. Kriging requires a variogram model to compute variable values for any possible sampling interval. The variogram functionality in conjunction with kriging allows us to estimate the accuracy with which a value at an unsampled location can be predicted given the sample values at other locations [28–30].

### 3.2. Geostatistical Spatial Analysis

The experimental variogram characterizes the spatial variability in the measured data is used in kriging to produce soil moisture maps and estimate values at unsampled locations. One of the major issues in variogram analysis is the selection of total lag distance for variogram fitting to experimental data [31]. As the separation distance increases, after half of the total separation distance, the variogram starts to decompose at larger separation distances due to the reduced availability of pairs. Thus to obtain robust estimation of the variogram, we ignored pairs at larger separation distances that usually have smaller variance. The separation distance is selected based on the criterion that 95% pairs should have been used for variogram model fitting. The effect of removing data pairs at larger separation distance significantly improves variogram model fitting to the AGRMET and Oklahoma Mesonet soil moisture data.

The fitting of the appropriate model to the experimental variogram data is another important step in geostatistical analysis. The fitting of the model can be done by personal judgment, or an automatic procedure can be followed to reduce subjectivity and to increase reproducibility. Different models can be fitted to the experimental semi-variance values. The most commonly used models, linear, spherical, exponential and Gaussian, were chosen to fit the experimental semi-variance plot, generated from soil moisture data, using least squares curve fitting. The elements of each variogram model and the regression coefficient R^2^ of the fitting procedure were determined. The model with the higher value of R^2^ was selected as an appropriate model to represent the sample variogram. The theoretical variogram model (Gaussian, spherical, exponential, or linear) that best fits the experimental variogram of AGRMET and Oklahoma Mesonet data was selected for soil moisture mapping using the block Kriging technique [26].

A jack-knifing method [32] was applied to evaluate the performance of the kriging technique at different locations of the Oklahoma Mesonet when compared with true soil moisture values. The jack-knifing method is a process where a small set of stations that have been selected for the comparison study are left out and not used to generate the variogram and kriging soil moisture estimates. This method ensures unbiased validation of the kriging estimates by examining and quantifying the errors associated with estimating soil moisture using the kriging process. The estimated values at the selected Oklahoma Mesonet stations were obtained by creating a variogram and kriging estimate using information from the rest of the sites. This procedure provided measured and estimated values for each sample location, so that actual estimation errors could be computed and compared.

### 3.3. Oklahoma Mesonet Data Screening and Quality Control

Because of its extensive network the Oklahoma Mesonet has a sufficient number of stations and spatial extent is idea for large scale spatial analysis using geostatistical techniques. While analyzing the Oklahoma Mesonet soil moisture data, we observed that some of the data at some of the sites were either unrealistic or had little soil moisture change throughout the month of study. Some of the sensors did not respond to high precipitation events that occurred during the study period. This could be attributed to saturated soil, sensors malfunctioning underground or calibration issues. Therefore, it is a prerequisite that such sites (sensors) be removed from further spatial analysis.

In this study, Soil Moisture-Precipitation Quality Control (SMPQC) based on the autocorrelation of change in soil moisture with respect to precipitation events was tested. The SMPQC test was carried out for all Oklahoma Mesonet sites to estimate auto-correlation values for each site. A threshold limit of autocorrelation value was selected based on detailed temporal analysis of each sites to eliminate the non-responsive (to the precipitation) soil moisture sites.

The locations of filtered sites after applying the SMPQC test to all Oklahoma Mesonet sites are shown in Figure 2. For sake of simplicity using geostatistical analysis, the sites in the panhandle area of Oklahoma were not selected. The application of SMPQC test has been limited by available soil moisture data (30 days) for each Oklahoma Mesonet location. The average number of precipitation events occurred at each site varies between 2 to 7. However, some of the precipitation events have less than 5 mm of total rainfall during events, which may not lead to changes in soil moisture measured at 5 cm below the surface. Hence such events were not considered while testing the SMPQC. A more comprehensive evaluation of this SMPQC test would require additional months of soil moisture data.

## 4. Results and Discussion

### 4.1. In Situ and Model Soil Moisture Comparison (Oklahoma Mesonet vs. AGRMET)

**Figure 4.**
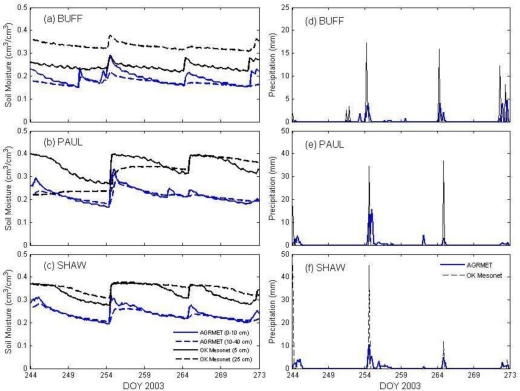
Soil moisture and precipitation comparisons between *in situ* Oklahoma Mesonet and AGRMET data for BUFF (a, d), PAUL (b, e), and SHAW (c, f) during September 2003.

Soil moisture and precipitation from Oklahoma Mesonet and AGRMET model were compared at three sites selected randomly based geographical locations. The Oklahoma Mesonet soil moisture values at 5 and 25 cm depths from: BUFF (0.5 miles SW of Buffalo, Latitude: 36°49′52″N, Longitude 99°38′27″W), PAUL (1.0 miles SSW of Pauls Valley Latitude 34°42′55″N, Longitude: 97°13′45″W), and SHAW (3.0 miles NNW of Shawnee, Latitude 35°21′53″N, Longitude 96°56′53″W) were compared with AGRMET data at 0–10 cm and 10–40 cm depth (Figure 4). The detailed characteristics of these sites can be found at the Oklahoma Mesonet website (http://www.mesonet.org).

Four significant rain events occurred during September (1.7 and 0.28 cm), the 11th (4.09 cm), 21st (3.68 cm), and 30th (0.15 cm). There is good soil moisture response for all of the events for both the station and AGRMET. At BUFF, four main rain events (Figure 4b) occurred during the month on September 6th (0.61 cm), 11th (2.44 cm), 21st (1.96 cm) and the 29–30th (1.68 and 0.99 cm). AGRMET showed good response to all four events, especially for the 0–10 cm depth. The *in situ* data response was muted; there is no response for the first event and some response at both 5 and 25 cm depth for the other precipitation events. The moisture difference observed for BUFF is due to a difference in rain between the model output and the station data.

Figure 4 shows the comparison, and the station measured greater amounts of precipitation than the model, though the AGRMET model data lagged by approximately 3–6 hours. It is possible that there was a heavy rain event during the last days of August. The ground may have started off saturated, so additional rain became run-off instead of increasing soil moisture. However, that cannot account for the lack of drying in between events. This leaves the possibility of unresolved station calibration issues, due to sensor hardware or incorrect calibration coefficients, or possibly equivalent soil texture errors within AGRMET model.

The AGRMET model output shows significant response to precipitation events but may have issues with the actual precipitation amounts being used to calculate the response. The AGRMET model’s response to precipitation events is justified to the soil moisture values. At, BUFF, AGRMET shows higher drying rate compared to MESONET sensors. Soil moisture from PAUL and SHAW shows better agreement with AGRMET soil moisture data (Figures 4b,c). Rain events are also comparable in timing, though the *in situ* rain amounts tend to be higher than AGRMET model (Figures 4d–f).

The total precipitation measured at BUFF, PAUL and SHAW stations are 42.4 mm, 97.7 mm, and 94.7 mm, as compared to the AGRMET precipitation of 79 mm, 101.3 mm and 122.75 mm, respectively. The BUFF station observed the largest difference in precipitation accumulation over the month. In terms of soil moisture, the average difference between AGRMET and Mesonet stations: BUFF, PAUL and SHAW observed are 5%, 12% and 9% of volumetric soil moisture. However, these errors much higher than 4% of average soil moisture, which is estimated over all 74 filtered Oklahoma Mesonet sites and 77 grid locations of AGRMET data. These randomly selected BUFF, PAUL and SHAW stations have higher soil moisture differences (as compared to AGRMET data) than do the remaining Mesonet station data average differences, but they are good representations of the common challenges inherent in the *in situ* data comparisons.

**Figure 5.**
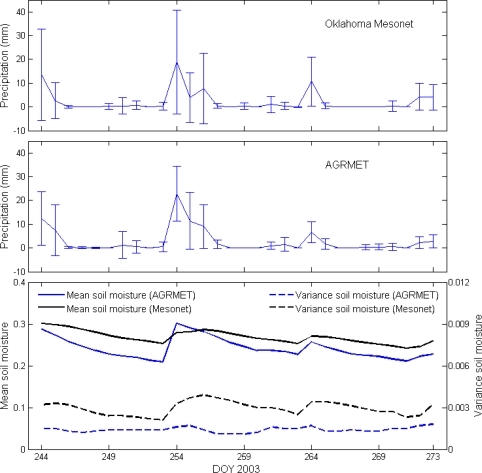
Mean and standard deviation of precipitation (a) observed by Oklahoma Mesonet, (b) AGRMET precipitation (c) soil moisture measured at Oklahoma Mesonet sites and derived from AGRMET model.

The comparison of the mean precipitation for all 74 filtered Oklahoma Mesonet sites and 77 grid locations of AGRMET data shows that the AGRMET model overestimates rainfall amount during large precipitation events and underestimates rainfall amount during smaller rainfall events (Figure 5a,b). The standard-deviations with respect to the mean of AGRMET precipitation measurements are smaller than *in situ* Oklahoma Mesonet data. This is due to smoothing effect resulting from the spatial resolution of the satellite data that is used as an input to the AGRMET model.

Mean soil moisture values are higher after precipitation events with higher variance being observed during wet periods after precipitation (Figure 5c). This could be due to spatially varying soil hydraulic properties creating differential infiltration rates during wet periods following rainfall, causing larger variation in soil moisture. Smaller variation is observed during dry periods where soil-related variability becomes minimal [33]. The AGRMET model underestimates the soil moisture compared to *in situ* measurements. However, the differences between average soil moisture values are smaller after precipitation or in wet soil conditions between both datasets. The variance of soil moisture increased during precipitation events for Oklahoma Mesonet data compared to more stable variance in the AGRMET data. Based on the trend (Figure 5c), it can be concluded that higher drying rates have been assumed within the AGRMET model.

### 4.2. Variogram Analysis of Soil Moisture

**Figure 6.**
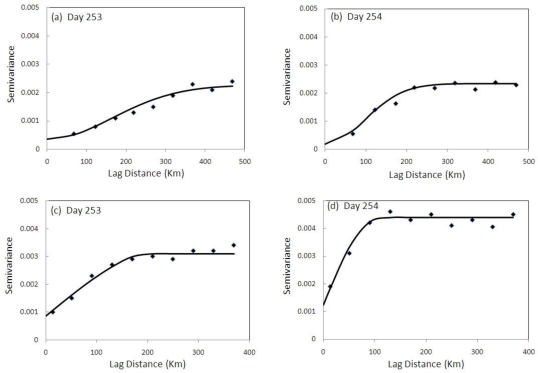
Soil moisture variograms of AGRMET (a-b) and Oklahoma Mesonet (c-d), where symbols are the experimental semi-variances and the solid lines show the fitted model. Variograms (a) and (c) are before precipitation event (253 day), and (b) and (d) after a precipitation event (254 day). Gaussian and Spherical models best fit the AGRMET and Oklahoma Mesonet soil moisture data, respectively.

The average distance between adjacent *in situ* (Oklahoma Mesonet network) soil moisture sites is 51 km, which is comparable to the grid resolution (47 km) of AGRMET soil moisture data. The variogram for daily average soil moisture values from Oklahoma Mesonet and AGRMET were produced. The characteristics of the variogram before and after precipitation events were shown in Figure 6. Based on the data, the Gaussian variogram model was the best fit for the AGRMET soil moisture data while the spherical model was better suited to the Oklahoma Mesonet soil moisture data. This is due to the fact that smoothing may have already occurred in AGRMET data due to its information source, *i.e.* Special Sensor Microwave/Imager (SSM/I), having a resolution of ∼50 km. Most of the variograms fit with non-zero nuggets.

The Oklahoma Mesonet variogram contains an outlier with exceedingly high semi-variance resulting from a large difference in soil moisture between the two closest pairs of observation PORT (Lat: 35°49′32″N, Long: 95°33′35″W) and HASK (Lat: 35°44′52″N, Long: 95°38′25″W); PERK (Lat: 35°59′55″N, Long: 97°2′53″W) and STIL (Lat: 36°7′15″N, Long: 97°5′42″W). Although these stations show appropriate variations in soil moisture, precipitation sensor calibration and soil type variation can significantly influence the semi-variance. Additionally, the number of samples at this small separation distance was not statistically significant as there were only two data points available. Similar observations for nearby samples were also made by Hollingsworth and Lönnberg [34].

Variograms were generated for all days of September 2003 for AGRMET and Oklahoma Mesonet soil moisture data. The largest change in variogram properties for AGRMET and Oklahoma Mesonet soil moisture data was observed after heavy precipitation on day 254 compared to day 253 (Figure 6). The comparison of decorrelation length and average soil moisture shows the effect of precipitation on change in decorrelation length (A_0_). The decorrelation length was higher for dry periods before precipitation and decreases with increasing soil moisture during and after precipitation events. Soil moisture decorrelation length is found to be higher than the precipitation decorrelation length, and is discussed in Haberlandt [35].

**Figure 7.**
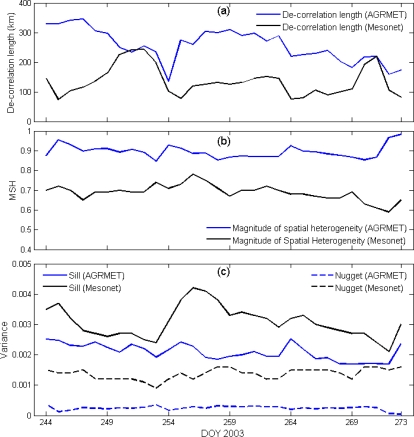
(a) Decorrelation lengths are higher for AGRMET compared to Oklahoma Mesonet soil moisture data, (b) Magnitude of spatial heterogeneity (MSH) is the ratio of Sill and Nugget for AGRMET and Oklahoma Mesonet soil moisture data. The MSH represents magnitude of spatial dependence which is higher during wet soil conditions, (c) Variogram elements (Sill and Nugget) show higher Sill (variance) and Nugget for Oklahoma Mesonet data compared to for AGRMET soil moisture data.

The variogram parameters (nugget, sill, and decorrelation length) have been estimated for each day at 0000 UTC of September 2003 for the AGRMET and Oklahoma Mesonet soil moisture data sets. The smallest decorrelation length as predicted by AGRMET was observed on day 254. Higher sill values are observed at higher soil moisture values (Figure 7). However, nugget values are almost constant over the time period. The spatial variability increases rapidly (as indicated by the reduction of decorrelation length) for higher values of average Oklahoma Mesonet soil moisture data. We observed that decreasing soil moisture reduced the spatial variability. However, decorrelation lengths can be different for similar soil moisture values, which could be influenced by differences in local or regional precipitation events. The sill (variance) of the variogram follows the trend of mean Oklahoma Mesonet soil moisture values (Figure 7), with higher soil moisture leading to an increase in sill. The nugget values are almost constant over the time period, particularly for AGRMET.

The time series comparison of decorrelation length estimated for AGRMET and Oklahoma Mesonet (Figure 7), shows that decorrelation length is higher in the case of AGRMET soil moisture data. However, the decorrelation length matches at two instances, specifically after precipitation events. The larger decorrelation in the case of AGRMET compared to Oklahoma Mesonet data is due to AGRMET’s spatial averaging versus the point sampling for the Oklahoma Mesonet data.

The magnitude of spatial heterogeneity (MSH) can be estimated as the proportion of total sample variation accounted for by spatially structured variation [28,36]. The MSH has been used widely to estimate the magnitude of spatial dependence that can be described by the variogram. As MSH approaches unity, a higher proportion of the total sample variance is spatially dependent over the separation distance examined. The MSH calculated using variogram parameters [C/(C + C_0_)] throughout the month for AGRMET and Oklahoma Mesonet data (Figure 7) were higher at wet soil conditions. This is because the precipitation-forced soil moisture patterns are at their strongest during wet events and have yet to be damped during the dry-down phase. The dry-down phase tends to diminish spatial patterns of soil moisture since each value slowly converges to lower soil moisture values that tend to be clustered at similar low soil moisture levels. However, if precipitation occurs in only part of the study area, higher values of MSH may not be observed.

### 4.3. Kriging Performance Assessment

**Figure 8.**
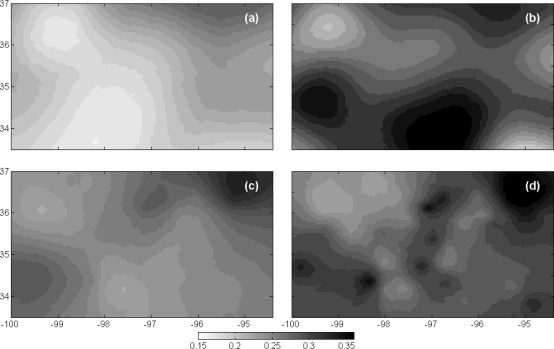
Kriged map of soil moisture for AGRMET data (a and b) and Mesonet data (c and d) generated using semi-variograms shown in Figure 6. Figures (a) and (c) are before precipitation event (253 day), and (b) and (d) for after precipitation event (253 day).

The validation of the kriging performance was carried out after analyzing the variogram characteristics. Soil moisture maps produced using kriging were compared with *in situ* soil moisture stations, which were not used in the variogram analysis. Contrasts were observed in variogram shape as well kriged soil moisture map before and after a rain event on day 254 (20 mm average over study area). Soil moisture maps (Figure 8) were generated at 5 km resolution from the variograms (Figure 6) for AGRMET and Oklahoma Mesonet data before and after a rain event. The kriged soil moisture maps show the different dynamics of soil moisture variation before and after precipitation events. The soil moisture spatial distributions after precipitation events show a marked change in the pattern, with an increase in spatial variation and also the appearance of isolated patches.

The spatial variability in terms of decorrelation length is reduced by 98 km from pre-rain to post rain times for AGRMET data. The effect of rainfall on the change in the variogram and soil moisture maps can be seen through the wet soil moisture conditions. In the soil moisture maps, higher spatial variability can be observed after the rainfall event for both datasets. The field sensors such as HASK and PORT or PERK and STIL are closer to each other when compared to other sensors in the network, yet show large difference (10–15%) in soil moisture values.

**Figure 9.**
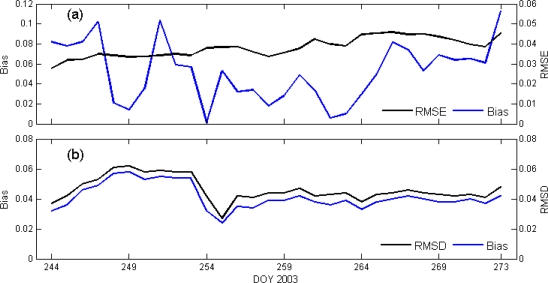
Kriging performance assessment (a) Bias and RMSE between kriged soil moisture map and ten Jackknifed site locations (b) Area average whole network, Root mean square difference (RMSD) and bias between kriged Mesonet and AGRMET soil moisture maps shows higher RMSD during dry period.

To test the performance of the kriging technique, 10 sites (15% of total sites) out of 74 sites were selected randomly distributed across the Oklahoma Mesonet area (Table 1). Thus, a list of measured values and kriged values was obtained for the set of stations, and the distribution of errors was analyzed. The true values (z) were compared with the kriged soil moisture (z*) using performance measures such as bias, root mean square error (RMSE) and correlation coefficient (R^2^). No specific trends in bias and RMSE, specific to the wet and dry periods were observed at the locations (Figure 9a). During the month, mostly positive biases were observed at KING, MARE, MAYR, and MEDI. The negative bias observed at KETC, MINC, NOWA, and OKEM. LAHO and OKMU sites were small. The average RMSE of 10 jackknifed sites was found to be 3.2% through September 2003 (Table 1). Larger RMSE (∼5.5%) were observed at KETC, MEDI and MIAM; and lower RMSE (about 1–1.5%) were observed at LAHO, MAYR and OKMU Mesonet sites. The RMSE values could potentially be lowered through use of a co-kriging analysis [26] by including precipitation as an additional variable.

The kriged soil moisture maps produced using *in situ* Oklahoma Mesonet data were used to (1) analyze kriging method to estimate soil moisture at unsampled location (Figure 9a), and (2) compare with AGRMET soil moisture data (Figure 9b). On temporal scale, root mean square of the error (RMSE) was estimated using kriged soil moisture map for 10 Jackknifed stations which were not used in kriged analysis (Figure 9a). The RMSE for Jackknifed stations were consistent and varies from 3% to 4% of volumetric soil moisture. However, bias is not consistent across the temporal scale.

Similarly, on temporal scale, the differences between Oklahoma Mesonet and AGRMET soil moisture maps produced using the kriging method were compared using average root mean square of the difference (RMSD) between the soil moil moisture values (Figure 9b). The average RMSD between kriged Oklahoma Mesonet and AGRMET soil moisture maps for the study area was 4.6% of soil moisture (Figure 9b). Higher RMSD was observed during drying period which could be due to an erroneous drying rate in the AGRMET model. Bias is lower than RMSD, though the study period follows a consistent trend with RMSD. Figure 9 shows clear distinguish between RMSE/RMSD and Bias due to local sites (Figure 9a) specific measurements compared with statewide area average (Figure 9b) calculations.

## 5. Summary and Conclusions

This study evaluates the statistical spatial structure of large-scale observed and modeled estimates of soil moisture under the pre and post precipitation event using a geostatistical approach. Independent *in situ* soil moisture measurements were compared with the AGRMET model output. Results indicate a tendency for the AGRMET precipitation input estimates to bias the model soil moisture results. This can result in entire rain events being omitted or added to the AGRMET output. When the AGRMET precipitation estimate is more realistic, the AGRMET soil moisture estimate improves. In addition, some Oklahoma Mesonet sites performed better than others as compared to *in situ* precipitation measurements. The variance of precipitation in AGRMET is observed to be smaller than *in situ* precipitation measurements from the Oklahoma Mesonet.

*In situ* measurement systems used in this study that were originally considered to be research-grade soil moisture networks were found to be susceptible to quality control issues. A simple test such as SMPQC for quality control of *in situ* data is necessary to eliminate the soil moisture sensors which did not respond well to precipitation. Some data networks experienced > 30% sensor failure rates using our more detailed quality control analysis procedures. Remaining quality-controlled data sets indicated that precipitation inputs were the primary cause of discrepancies between the AGRMET model output and in situ soil moisture measurements. However, in some circumstances soil texture and possibly other AGRMET model parameters or inputs are suspected to be the cause of inconsistent soil moisture output results. In addition, due to the spatial representation errors we do not expect perfect model versus in situ agreement, although application of downscaling methods may be able to partially mitigate these errors [37]. We expect that improved in situ sensor calibration and quality control methods would increase the reliably of the soil moisture measurements. Further, in such conditions, techniques such as kriging described in this study may mitigate some of the quality control errors with appropriate geostatistical information.

The characteristics of the variogram models are analyzed for dry and wet conditions using AGRMET and *in situ* Oklahoma Mesonet soil moisture data. Variogram structure changes primarily due to precipitation and drying of the soil surface. Spatial statistical behaviors of the AGRMET model output were smoother (as expected) than co-located *in situ* data “point” measurements. This indicates that spatial structures should be considered when using the *in situ* data as part of any future calibration/validation program. The analysis of the variogram structure of AGRMET and Oklahoma Mesonet data indicates that the decorrelation length is higher for AGRMET compared to Oklahoma Mesonet data. This could be due to the smoothing effect in soil moisture estimation using AGRMET model, as higher smoothing leads to larger decorrelation length. Pre-precipitation regimes were found to have a higher decorrelation length than post-precipitation regimes indicating that precipitation storm-scales drive soil moisture spatial structures. This large scale soil moisture variability information is important to gain confidence in the AGRMET model performance, as well as integration with other large-scale (satellite) data assimilation systems. The comparison between kriged soil moisture maps of AGRMET and Oklahoma Mesonet data were correlated to precipitation event timing, thus indicating areas of possible AGRMET improvements related to precipitation input forcing.

The multivariate kriging techniques such as: Co-kriging, External drift kriging, Indicator kriging (IK), or External drift indicator kriging (EDIK), can be used to improve the soil moisture accuracy using additional information such as topography, soil texture, and land cover. Future work should also perform more detailed spatial analysis to automate the detection of false signals within a dispersed soil moisture network. This type of spatial analysis is of immediate use to help determine the average distance between the soil moisture stations for establishing large soil moisture network such as: U.S. Climate Reference Network (USCRN). In addition, the quantification of co-variances will be used to advance satellite data assimilation experiments [38]. It was beyond the scope of this work to implement the observational covariance information within the 4DVAR methodologies, but is a work in progress. The results contained in this study are fundamental to the performance and behaviors of future 4 DVAR assimilation for soil moisture retrieval using WindSat and future NPOESS satellite data, and will also direct future research activities toward areas requiring additional improvements.

## Figures and Tables

**Table 1.** This table shows the performance of Kriging in terms of volumetric soil moisture at each jack-knifed Mesonet site for September 2003.

**Site Name**	**Latitude**	**Longitude**	**Bias**	**RMSE**	**Correlation Coefficient**

KETC	34.529	−97.765	−0.059	0.060	0.81
KING	35.881	−97.911	+0.027	0.030	0.97
LAHO	36.384	−98.111	−0.006	0.008	0.94
MARE	36.064	−97.213	+0.047	0.048	0.86
MAYR	36.987	−99.011	+0.012	0.013	0.80
MEDI	34.729	−98.567	+0.060	0.061	0.94
MINC	35.272	−97.956	−0.023	0.026	0.86
NOWA	36.744	−95.608	−0.027	0.032	0.46
OKEM	35.432	−96.263	−0.025	0.026	0.86
OKMU	35.581	−95.915	−0.011	0.013	0.91
**All Sites**	--	--	--	**0.032**	**0.84**
